# Bis(benzoato-κ^2^ *O*,*O*′)(1,10-phenanthroline-κ^2^ *N*,*N*′)lead(II) benzoic acid mono­solvate

**DOI:** 10.1107/S1600536810048725

**Published:** 2010-11-27

**Authors:** Jun Dai, Juan Yang, Jiantong Li

**Affiliations:** a Institute of Safety Science and Engineering, Henan Polytechnic University, Jiaozuo 454003, People’s Republic of China; b Department of Physical Chemistry, Henan Polytechnic University, Jiaozuo 454003, People’s Republic of China

## Abstract

The reaction of lead acetate, benzoic acid and 1,10-phenanthroline (phen) in aqueous solution yielded the title complex, [Pb(C_7_H_5_O_2_)_2_(C_12_H_8_N_2_)]·C_7_H_6_O_2_. In the crystal, the Pb^II^ ion is hexa­coordinated by two N atoms from one 1,10-phenanthroline ligand and four O atoms from two chelate benzoate anions. If the second benzoate ligand is treated as one coordination site, the overall coordination may be represented as a distorted pseudo-square pyramid. An inter­molecular O—H⋯O hydrogen bond links the solvent benzoic acid mol­ecule with a metal-coordinated benzoate ligand. The shortest Pb⋯Pb distance is 3.864 (4) Å, indicating a weak metal–metal inter­action. Two complex mol­ecules related by an inversion centre form dimeric units *via* Pb⋯O inter­actions of 3.206 (4) Å.

## Related literature

For general background to the applications of complexes containing Pb(II) ions, see: Fan & Zhu (2006 ▶); Hamilton *et al.* (2004 ▶); Alvarado *et al.* (2005 ▶). For the use of aromatic carboxyl­ates and the phenanthroline ligand in the preparation of metal complexes, see: Wang *et al.* (2006 ▶); Yang *et al.* (2010 ▶).
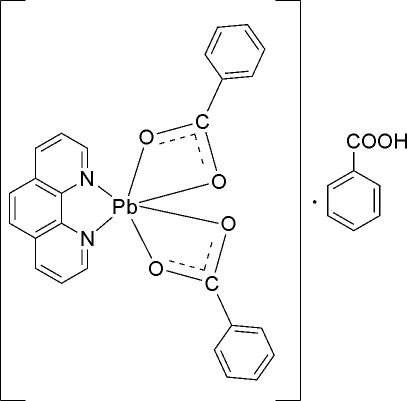


## Experimental

### 

#### Crystal data

[Pb(C_7_H_5_O_2_)_2_(C_12_H_8_N_2_)]·C_7_H_6_O_2_
*M* *_r_* = 751.73
Triclinic, 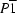

*a* = 10.0725 (8) Å
*b* = 10.5697 (8) Å
*c* = 15.5477 (17) Å
α = 93.414 (2)°
β = 102.836 (2)°
γ = 117.972 (1)°
*V* = 1399.3 (2) Å^3^
*Z* = 2
Mo *K*α radiation
μ = 6.08 mm^−1^
*T* = 296 K
0.26 × 0.18 × 0.15 mm

#### Data collection

Bruker APEXII CCD area-detector diffractometer
Absorption correction: multi-scan (*SADABS*; Bruker, 2007 ▶) *T*_min_ = 0.263, *T*_max_ = 0.582
8277 measured reflections
5710 independent reflections
4950 reflections with *I* > 2σ(*I*)
*R*_int_ = 0.020

#### Refinement

*R*[*F* ^2^ > 2σ(*F* ^2^)] = 0.029
*wR*(*F* ^2^) = 0.074
*S* = 1.02
5710 reflections
379 parameters
H-atom parameters constrained
Δρ_max_ = 0.73 e Å^−3^
Δρ_min_ = −0.73 e Å^−3^

### 

Data collection: *APEX2* (Bruker, 2007 ▶); cell refinement: *SAINT* (Bruker, 2007 ▶); data reduction: *SAINT*; program(s) used to solve structure: *SHELXS97* (Sheldrick, 2008 ▶); program(s) used to refine structure: *SHELXL97* (Sheldrick, 2008 ▶); molecular graphics: *SHELXTL* (Sheldrick, 2008 ▶); software used to prepare material for publication: *SHELXTL*.

## Supplementary Material

Crystal structure: contains datablocks global, I. DOI: 10.1107/S1600536810048725/bt5413sup1.cif

Structure factors: contains datablocks I. DOI: 10.1107/S1600536810048725/bt5413Isup2.hkl

Additional supplementary materials:  crystallographic information; 3D view; checkCIF report

## Figures and Tables

**Table 1:** Selected bond lengths (Å)

Pb1—O1	2.337 (3)
Pb1—O3	2.361 (4)
Pb1—N2	2.564 (3)
Pb1—N1	2.632 (4)
Pb1—O2	2.822 (3)
Pb1—O4	2.928 (4)

**Table 2:** Hydrogen-bond geometry (Å, °)

*D*—H⋯*A*	*D*—H	H⋯*A*	*D*⋯*A*	*D*—H⋯*A*
O5—H5⋯O2	0.82	1.94	2.654 (5)	145

**Fig. 1.**
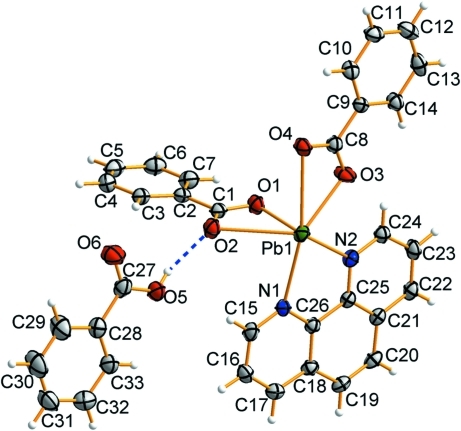
The asymmetric unit of the title complex, showing displacement ellipsoids at the 30% probability level and the atom-labeling scheme. The H-bond is shown as dashed lines in blue.

**Fig. 2.**
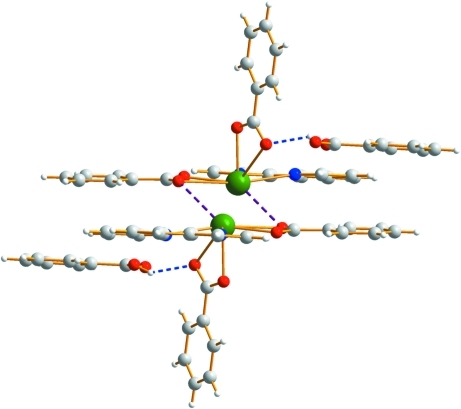
The dimer structure of complexes, formed by the weak intermolecular Pb···O interactions (dashed lines in violet).

## supplementary crystallographic information

### Comment

Complexes containing Pb(II) ions have recently attracted considerable interest not only because of the variety of their architectures, but also because of their potential applications, especially in environmental protection and in systems with different biological properties (Fan & Zhu, 2006; Hamilton *et al.*, 2004; Alvarado *et al.*, 2005). As an important family of bidentate O-donor ligands, aromatic carboxylates have been extensively employed in the preparation of metal complexes of various structural topologies (Wang *et al.*, 2006; Yang *et al.*, 2010).

The asymmetric unit of the title complex, [Pb(C_7_H_5_O_2_)_2_(C_12_H_8_N_2_)] &bull;(C_7_H_6_O_2_), contains a Pb^II^ cation, two benzoate ligands, one 1,10-phenanthroline (phen) ligand and one free benzoic acid molecule, as illustrated in Fig.1. In the crystal, the Pb^II^ ion is hexacoordinated by two N atoms from one phen ligand and four O atoms from two chelate benzoate anions.The O atoms from one of carboxylate ligands (O3 and O4) are almost coplanar with the N atoms of the phen-ligand (N1 and N2)[dihedral angle of 10.49 (12)°]. Therefore, if we consider that the second carboxylate ligand occupies just one coordination site, the coordination environment of Pb^II^ ion may be described as pseudo-square-pyramidal. The intermolecular hydrogen bond exist between the carboxyl H atom of solvent benzoic acid and metal-coordinated carboxylate O atom. The inter-distance of Pb···Pb^i^ [^i^ 1 - *x*, 1 - *y*, 1 - *z*] is 3.864 (4) Å, indicating the weak metal-metal interaction. The complex molecules related by inversion center are organized into dimeric units *via* a pair of Pb···O interactions of 3.206 (4) Å (Fig.2) and stacking inteactions between phen and benzoate ligands, with the shortest centroid–centroid distance between their planes of 3.521 (5) Å.

### Experimental

A mixture of Pb(CH_3_COO)_2_^.^ 3H_2_O (0.05 g, 0.13 mmol), benzoic acid (0.102 g, 0.84 mmol), 1,10-phenanthroline (0.083 g, 0.41 mmol) and distilled water (10 ml) was sealed in a 25 ml Teflon-lined stainless autoclave. The mixture was heated at 403 K for 6 days to give the colorless crystals suitable for X-ray diffraction analysis.

### Refinement

All H atoms bound to C atoms were placed in calculated positions and treated in a riding-model approximation, with C—H = 0.93 Å and *U*_iso_ (H) = 1.2 *U*_eq_(C). The   carboxylic H atom was located in a difference Fourier map. Nevertheless, it was treated as riding  with an idealized distance of O—H = 0.82 Å and *U*_iso_ (H) = 1.2 *U*_eq_(O).

### Crystal data

**Table:**

[Pb(C_7_H_5_O_2_)_2_(C_12_H_8_N_2_)]·C_7_H_6_O_2_	*Z* = 2
*M**_r_* = 751.73	*F*(000) = 732
Triclinic, *P*1	*D*_x_ = 1.784 Mg m^−^^3^
Hall symbol: -P 1	Mo *K*α radiation, λ = 0.71073 Å
*a* = 10.0725 (8) Å	Cell parameters from 3689 reflections
*b* = 10.5697 (8) Å	θ = 1.4–26.5°
*c* = 15.5477 (17) Å	µ = 6.08 mm^−^^1^
α = 93.414 (2)°	*T* = 296 K
β = 102.836 (2)°	Prism, colorless
γ = 117.972 (1)°	0.26 × 0.18 × 0.15 mm
*V* = 1399.3 (2) Å^3^	

### Data collection

**Table:**

Bruker APEXII CCD area-detector diffractometer	5710 independent reflections
Radiation source: fine-focus sealed tube	4950 reflections with *I* > 2σ(*I*)
graphite	*R*_int_ = 0.020
ω scans	θ_max_ = 26.5°, θ_min_ = 1.4°
Absorption correction: multi-scan (*SADABS*; Bruker, 2007)	*h* = −12→12
*T*_min_ = 0.263, *T*_max_ = 0.582	*k* = −10→13
8277 measured reflections	*l* = −15→19

### Refinement

**Table:**

Refinement on *F*^2^	Primary atom site location: structure-invariant direct methods
Least-squares matrix: full	Secondary atom site location: difference Fourier map
*R*[*F*^2^ > 2σ(*F*^2^)] = 0.029	Hydrogen site location: inferred from neighbouring sites
*wR*(*F*^2^) = 0.074	H-atom parameters constrained
*S* = 1.02	*w* = 1/[σ^2^(*F*_o_^2^) + (0.038*P*)^2^] where *P* = (*F*_o_^2^ + 2*F*_c_^2^)/3
5710 reflections	(Δ/σ)_max_ = 0.001
379 parameters	Δρ_max_ = 0.73 e Å^−^^3^
0 restraints	Δρ_min_ = −0.73 e Å^−^^3^

### Fractional atomic coordinates and isotropic or equivalent isotropic displacement parameters (Å^2^)

**Table:**

	*x*	*y*	*z*	*U*_iso_*/*U*_eq_	
Pb1	0.595637 (19)	0.391099 (18)	0.465657 (12)	0.04103 (7)	
O1	0.7152 (4)	0.4830 (4)	0.3537 (2)	0.0547 (9)	
O2	0.4876 (4)	0.4755 (4)	0.3081 (3)	0.0577 (9)	
O3	0.8489 (4)	0.5402 (4)	0.5653 (3)	0.0659 (11)	
O4	0.7626 (4)	0.6979 (4)	0.5547 (3)	0.0567 (9)	
O5	0.2159 (4)	0.3215 (4)	0.1819 (3)	0.0696 (11)	
H5	0.3118	0.3665	0.2014	0.084*	
O6	0.1681 (6)	0.5034 (5)	0.1686 (4)	0.0944 (15)	
N1	0.4405 (4)	0.1384 (4)	0.3537 (3)	0.0416 (9)	
N2	0.7216 (4)	0.2286 (4)	0.4793 (3)	0.0393 (8)	
C1	0.6176 (6)	0.5023 (5)	0.2969 (3)	0.0452 (11)	
C2	0.6615 (5)	0.5607 (5)	0.2168 (3)	0.0424 (11)	
C3	0.5676 (6)	0.5993 (5)	0.1582 (3)	0.0488 (12)	
H3A	0.4771	0.5908	0.1695	0.059*	
C4	0.6080 (7)	0.6507 (6)	0.0828 (4)	0.0582 (13)	
H4A	0.5446	0.6765	0.0435	0.070*	
C5	0.7400 (7)	0.6634 (6)	0.0660 (4)	0.0632 (15)	
H5A	0.7654	0.6965	0.0147	0.076*	
C6	0.8358 (7)	0.6282 (6)	0.1236 (4)	0.0607 (14)	
H6A	0.9275	0.6402	0.1123	0.073*	
C7	0.7965 (6)	0.5747 (6)	0.1985 (4)	0.0563 (13)	
H7A	0.8602	0.5481	0.2367	0.068*	
C8	0.8635 (6)	0.6642 (5)	0.5888 (3)	0.0444 (11)	
C9	1.0096 (5)	0.7720 (5)	0.6587 (3)	0.0385 (10)	
C10	1.0375 (6)	0.9115 (6)	0.6866 (3)	0.0482 (12)	
H10A	0.9629	0.9374	0.6620	0.058*	
C11	1.1724 (6)	1.0110 (6)	0.7493 (4)	0.0569 (13)	
H11A	1.1885	1.1038	0.7675	0.068*	
C12	1.2841 (6)	0.9756 (7)	0.7857 (4)	0.0644 (15)	
H12A	1.3764	1.0442	0.8282	0.077*	
C13	1.2596 (6)	0.8381 (7)	0.7592 (4)	0.0623 (15)	
H13A	1.3351	0.8134	0.7842	0.075*	
C14	1.1226 (6)	0.7364 (6)	0.6955 (3)	0.0484 (12)	
H14A	1.1067	0.6437	0.6773	0.058*	
C15	0.3050 (6)	0.0935 (6)	0.2932 (4)	0.0534 (13)	
H15A	0.2721	0.1612	0.2819	0.064*	
C16	0.2095 (6)	−0.0469 (6)	0.2458 (4)	0.0574 (14)	
H16A	0.1150	−0.0730	0.2037	0.069*	
C17	0.2566 (6)	−0.1472 (5)	0.2620 (3)	0.0504 (12)	
H17A	0.1938	−0.2429	0.2306	0.060*	
C18	0.3980 (5)	−0.1070 (5)	0.3252 (3)	0.0421 (10)	
C19	0.4560 (6)	−0.2050 (5)	0.3430 (3)	0.0457 (11)	
H19A	0.3956	−0.3019	0.3135	0.055*	
C20	0.5954 (6)	−0.1606 (5)	0.4015 (3)	0.0452 (11)	
H20A	0.6318	−0.2262	0.4111	0.054*	
C21	0.6893 (5)	−0.0133 (5)	0.4495 (3)	0.0376 (10)	
C22	0.8359 (6)	0.0362 (5)	0.5108 (3)	0.0451 (11)	
H22A	0.8740	−0.0278	0.5224	0.054*	
C23	0.9227 (6)	0.1780 (6)	0.5534 (4)	0.0524 (12)	
H23A	1.0210	0.2123	0.5936	0.063*	
C24	0.8615 (5)	0.2722 (5)	0.5357 (3)	0.0442 (11)	
H24A	0.9218	0.3693	0.5649	0.053*	
C25	0.6365 (5)	0.0866 (5)	0.4351 (3)	0.0355 (9)	
C26	0.4878 (5)	0.0390 (5)	0.3695 (3)	0.0371 (10)	
C27	0.1292 (6)	0.3771 (7)	0.1484 (4)	0.0581 (14)	
C28	−0.0215 (6)	0.2676 (6)	0.0814 (4)	0.0506 (12)	
C29	−0.1214 (8)	0.3134 (8)	0.0389 (5)	0.083 (2)	
H29A	−0.0936	0.4112	0.0515	0.100*	
C30	−0.2625 (9)	0.2159 (10)	−0.0223 (5)	0.102 (3)	
H30A	−0.3299	0.2480	−0.0499	0.122*	
C31	−0.3037 (8)	0.0742 (9)	−0.0426 (5)	0.089 (2)	
H31A	−0.3990	0.0085	−0.0839	0.107*	
C32	−0.2035 (9)	0.0287 (7)	−0.0015 (5)	0.084 (2)	
H32A	−0.2310	−0.0688	−0.0155	0.100*	
C33	−0.0624 (7)	0.1247 (6)	0.0603 (4)	0.0641 (15)	
H33A	0.0048	0.0920	0.0875	0.077*	

### Atomic displacement parameters (Å^2^)

**Table:**

	*U*^11^	*U*^22^	*U*^33^	*U*^12^	*U*^13^	*U*^23^
Pb1	0.03974 (10)	0.03631 (11)	0.05214 (12)	0.02321 (8)	0.01173 (8)	0.01013 (8)
O1	0.0413 (19)	0.064 (2)	0.061 (2)	0.0280 (17)	0.0112 (17)	0.0219 (18)
O2	0.045 (2)	0.068 (2)	0.066 (2)	0.0305 (18)	0.0171 (17)	0.0281 (19)
O3	0.058 (2)	0.042 (2)	0.085 (3)	0.0297 (18)	−0.011 (2)	−0.0138 (19)
O4	0.0458 (19)	0.050 (2)	0.068 (2)	0.0275 (17)	−0.0041 (17)	0.0032 (18)
O5	0.049 (2)	0.064 (2)	0.088 (3)	0.0263 (19)	0.005 (2)	0.028 (2)
O6	0.086 (3)	0.062 (3)	0.118 (4)	0.040 (3)	−0.007 (3)	−0.003 (3)
N1	0.038 (2)	0.040 (2)	0.046 (2)	0.0210 (18)	0.0070 (17)	0.0083 (18)
N2	0.041 (2)	0.040 (2)	0.045 (2)	0.0264 (18)	0.0130 (18)	0.0077 (17)
C1	0.041 (3)	0.032 (2)	0.053 (3)	0.015 (2)	0.006 (2)	0.004 (2)
C2	0.040 (2)	0.032 (2)	0.044 (3)	0.013 (2)	0.006 (2)	0.002 (2)
C3	0.045 (3)	0.041 (3)	0.053 (3)	0.018 (2)	0.010 (2)	0.008 (2)
C4	0.062 (3)	0.054 (3)	0.049 (3)	0.025 (3)	0.006 (3)	0.011 (3)
C5	0.074 (4)	0.047 (3)	0.056 (3)	0.018 (3)	0.024 (3)	0.006 (3)
C6	0.057 (3)	0.057 (3)	0.068 (4)	0.024 (3)	0.025 (3)	0.008 (3)
C7	0.050 (3)	0.049 (3)	0.070 (4)	0.024 (3)	0.017 (3)	0.010 (3)
C8	0.046 (3)	0.046 (3)	0.043 (3)	0.026 (2)	0.008 (2)	0.009 (2)
C9	0.035 (2)	0.035 (2)	0.041 (3)	0.0148 (19)	0.0085 (19)	0.006 (2)
C10	0.055 (3)	0.053 (3)	0.042 (3)	0.033 (3)	0.011 (2)	0.005 (2)
C11	0.058 (3)	0.045 (3)	0.052 (3)	0.017 (3)	0.011 (3)	−0.002 (2)
C12	0.046 (3)	0.067 (4)	0.051 (3)	0.010 (3)	0.007 (3)	−0.005 (3)
C13	0.046 (3)	0.089 (5)	0.053 (3)	0.037 (3)	0.008 (3)	0.015 (3)
C14	0.045 (3)	0.052 (3)	0.049 (3)	0.027 (2)	0.009 (2)	0.008 (2)
C15	0.047 (3)	0.050 (3)	0.067 (4)	0.029 (3)	0.009 (3)	0.021 (3)
C16	0.048 (3)	0.056 (3)	0.059 (3)	0.025 (3)	−0.001 (3)	0.007 (3)
C17	0.048 (3)	0.039 (3)	0.054 (3)	0.016 (2)	0.009 (2)	0.004 (2)
C18	0.039 (2)	0.040 (3)	0.048 (3)	0.019 (2)	0.015 (2)	0.010 (2)
C19	0.054 (3)	0.033 (2)	0.055 (3)	0.022 (2)	0.022 (2)	0.012 (2)
C20	0.055 (3)	0.041 (3)	0.056 (3)	0.031 (2)	0.027 (2)	0.016 (2)
C21	0.042 (2)	0.038 (2)	0.040 (2)	0.023 (2)	0.017 (2)	0.013 (2)
C22	0.046 (3)	0.049 (3)	0.053 (3)	0.033 (2)	0.016 (2)	0.016 (2)
C23	0.045 (3)	0.062 (3)	0.055 (3)	0.034 (3)	0.003 (2)	0.012 (3)
C24	0.043 (3)	0.046 (3)	0.044 (3)	0.024 (2)	0.009 (2)	0.009 (2)
C25	0.038 (2)	0.037 (2)	0.038 (2)	0.021 (2)	0.0147 (19)	0.0129 (19)
C26	0.037 (2)	0.038 (2)	0.039 (2)	0.020 (2)	0.0110 (19)	0.0096 (19)
C27	0.052 (3)	0.064 (4)	0.068 (4)	0.033 (3)	0.021 (3)	0.028 (3)
C28	0.051 (3)	0.060 (3)	0.053 (3)	0.035 (3)	0.019 (2)	0.014 (3)
C29	0.082 (5)	0.087 (5)	0.092 (5)	0.062 (4)	0.003 (4)	0.000 (4)
C30	0.083 (5)	0.118 (7)	0.108 (6)	0.071 (5)	−0.009 (5)	0.004 (5)
C31	0.061 (4)	0.100 (6)	0.076 (5)	0.029 (4)	−0.006 (3)	0.006 (4)
C32	0.087 (5)	0.059 (4)	0.084 (5)	0.025 (4)	0.012 (4)	0.011 (4)
C33	0.055 (3)	0.058 (4)	0.074 (4)	0.026 (3)	0.010 (3)	0.019 (3)

### Geometric parameters (Å, °)

**Table:**

Pb1—O1	2.337 (3)	C12—H12A	0.9300
Pb1—O3	2.361 (4)	C13—C14	1.384 (7)
Pb1—N2	2.564 (3)	C13—H13A	0.9300
Pb1—N1	2.632 (4)	C14—H14A	0.9300
Pb1—O2	2.822 (3)	C15—C16	1.373 (7)
Pb1—O4	2.928 (4)	C15—H15A	0.9300
O1—C1	1.269 (6)	C16—C17	1.365 (7)
O2—C1	1.259 (6)	C16—H16A	0.9300
O3—C8	1.269 (5)	C17—C18	1.391 (7)
O4—C8	1.250 (5)	C17—H17A	0.9300
O5—C27	1.303 (6)	C18—C26	1.401 (6)
O5—H5	0.8200	C18—C19	1.422 (6)
O6—C27	1.199 (6)	C19—C20	1.335 (7)
N1—C15	1.320 (6)	C19—H19A	0.9300
N1—C26	1.356 (6)	C20—C21	1.429 (6)
N2—C24	1.327 (6)	C20—H20A	0.9300
N2—C25	1.367 (5)	C21—C22	1.397 (6)
C1—C2	1.485 (7)	C21—C25	1.397 (6)
C2—C3	1.384 (7)	C22—C23	1.359 (8)
C2—C7	1.393 (7)	C22—H22A	0.9300
C3—C4	1.384 (7)	C23—C24	1.407 (7)
C3—H3A	0.9300	C23—H23A	0.9300
C4—C5	1.358 (8)	C24—H24A	0.9300
C4—H4A	0.9300	C25—C26	1.445 (6)
C5—C6	1.365 (8)	C27—C28	1.495 (8)
C5—H5A	0.9300	C28—C33	1.363 (7)
C6—C7	1.381 (8)	C28—C29	1.372 (8)
C6—H6A	0.9300	C29—C30	1.377 (10)
C7—H7A	0.9300	C29—H29A	0.9300
C8—C9	1.488 (7)	C30—C31	1.348 (10)
C9—C14	1.380 (6)	C30—H30A	0.9300
C9—C10	1.385 (6)	C31—C32	1.365 (10)
C10—C11	1.361 (7)	C31—H31A	0.9300
C10—H10A	0.9300	C32—C33	1.377 (9)
C11—C12	1.366 (8)	C32—H32A	0.9300
C11—H11A	0.9300	C33—H33A	0.9300
C12—C13	1.374 (8)		
			
O1—Pb1—O3	84.82 (14)	C9—C14—H14A	119.9
O1—Pb1—N2	88.82 (12)	C13—C14—H14A	119.9
O3—Pb1—N2	75.08 (11)	N1—C15—C16	124.2 (5)
O1—Pb1—N1	85.71 (12)	N1—C15—H15A	117.9
O3—Pb1—N1	137.85 (11)	C16—C15—H15A	117.9
N2—Pb1—N1	63.74 (12)	C17—C16—C15	118.4 (5)
O1—Pb1—O2	49.51 (10)	C17—C16—H16A	120.8
O3—Pb1—O2	121.87 (13)	C15—C16—H16A	120.8
N2—Pb1—O2	127.06 (11)	C16—C17—C18	120.3 (5)
N1—Pb1—O2	79.89 (11)	C16—C17—H17A	119.8
C1—O1—Pb1	105.9 (3)	C18—C17—H17A	119.8
C1—O2—Pb1	83.1 (3)	C17—C18—C26	117.1 (4)
C8—O3—Pb1	107.8 (3)	C17—C18—C19	123.0 (4)
C27—O5—H5	125.4	C26—C18—C19	119.8 (4)
C15—N1—C26	117.5 (4)	C20—C19—C18	121.2 (4)
C15—N1—Pb1	123.7 (3)	C20—C19—H19A	119.4
C26—N1—Pb1	117.7 (3)	C18—C19—H19A	119.4
C24—N2—C25	117.8 (4)	C19—C20—C21	120.7 (4)
C24—N2—Pb1	122.2 (3)	C19—C20—H20A	119.6
C25—N2—Pb1	119.5 (3)	C21—C20—H20A	119.6
O2—C1—O1	121.4 (5)	C22—C21—C25	117.9 (4)
O2—C1—C2	120.2 (4)	C22—C21—C20	121.9 (4)
O1—C1—C2	118.4 (4)	C25—C21—C20	120.2 (4)
C3—C2—C7	118.8 (5)	C23—C22—C21	119.9 (4)
C3—C2—C1	120.7 (4)	C23—C22—H22A	120.1
C7—C2—C1	120.4 (4)	C21—C22—H22A	120.1
C2—C3—C4	120.1 (5)	C22—C23—C24	119.0 (5)
C2—C3—H3A	119.9	C22—C23—H23A	120.5
C4—C3—H3A	119.9	C24—C23—H23A	120.5
C5—C4—C3	120.2 (5)	N2—C24—C23	122.9 (5)
C5—C4—H4A	119.9	N2—C24—H24A	118.6
C3—C4—H4A	119.9	C23—C24—H24A	118.6
C4—C5—C6	120.8 (5)	N2—C25—C21	122.5 (4)
C4—C5—H5A	119.6	N2—C25—C26	118.7 (4)
C6—C5—H5A	119.6	C21—C25—C26	118.8 (4)
C5—C6—C7	120.0 (5)	N1—C26—C18	122.5 (4)
C5—C6—H6A	120.0	N1—C26—C25	118.2 (4)
C7—C6—H6A	120.0	C18—C26—C25	119.2 (4)
C6—C7—C2	120.0 (5)	O6—C27—O5	123.2 (6)
C6—C7—H7A	120.0	O6—C27—C28	124.0 (5)
C2—C7—H7A	120.0	O5—C27—C28	112.8 (5)
O4—C8—O3	122.7 (5)	C33—C28—C29	118.9 (6)
O4—C8—C9	120.2 (4)	C33—C28—C27	122.5 (5)
O3—C8—C9	117.0 (4)	C29—C28—C27	118.6 (5)
C14—C9—C10	118.4 (4)	C28—C29—C30	120.6 (6)
C14—C9—C8	120.5 (4)	C28—C29—H29A	119.7
C10—C9—C8	121.0 (4)	C30—C29—H29A	119.7
C11—C10—C9	121.1 (5)	C31—C30—C29	120.5 (7)
C11—C10—H10A	119.5	C31—C30—H30A	119.7
C9—C10—H10A	119.5	C29—C30—H30A	119.7
C10—C11—C12	120.4 (5)	C30—C31—C32	119.0 (7)
C10—C11—H11A	119.8	C30—C31—H31A	120.5
C12—C11—H11A	119.8	C32—C31—H31A	120.5
C11—C12—C13	119.7 (5)	C31—C32—C33	121.2 (7)
C11—C12—H12A	120.1	C31—C32—H32A	119.4
C13—C12—H12A	120.1	C33—C32—H32A	119.4
C12—C13—C14	120.1 (5)	C28—C33—C32	119.8 (6)
C12—C13—H13A	120.0	C28—C33—H33A	120.1
C14—C13—H13A	120.0	C32—C33—H33A	120.1
C9—C14—C13	120.2 (5)		

### Hydrogen-bond geometry (Å, °)

**Table:**

*D*—H···*A*	*D*—H	H···*A*	*D*···*A*	*D*—H···*A*
O5—H5···O2	0.82	1.94	2.654 (5)	145
